# Addressing socio-cultural barriers to improve contraceptive use in Ethiopia

**DOI:** 10.12688/f1000research.163555.2

**Published:** 2025-06-17

**Authors:** Tadesse Shiferaw Chekol, Metasebia Tegegn Baheru

**Affiliations:** 1 One Health, Armauer Hansen Research Institute, Addis Ababa, 10005, Ethiopia; 2 College of Health Sciences, Addis Ababa University, Addis Ababa, Ethiopia

**Keywords:** Contraceptive use, socio-cultural barriers, Ethiopia

## Abstract

Ethiopia encounters significant socio-cultural barriers to contraceptive use, affecting maternal health, poverty reduction, and women’s empowerment. With a fertility rate of 4.6 children per woman and only 25.9% of women of reproductive age intending to use contraceptives, significant challenges remain. This policy brief analyzes data from the 2016 Ethiopian Demographic and Health Survey (EDHS) and 2019 Mini-EDHS, revealing key barriers such as fatalistic beliefs, religious prohibitions, postpartum amenorrhea, and spousal opposition. Socioeconomic factors, including wealth, literacy, and urban residency, greatly influence contraceptive use. Multivariable and structural equation modeling (SEM) analyses underscore the complex interplay of cultural norms, economic status, and individual autonomy in shaping contraceptive behavior. The result reveals both direct and indirect effects of socio-cultural factors such as fatalistic beliefs and religious prohibitions on contraceptive use. The findings of this brief provide direct policy relevance by identifying actionable pathways for engaging religious leaders, community influencers, and health workers in culturally responsive strategies to overcome socio-cultural barriers and improve contraceptive uptake. Findings recommend to enhance contraceptive uptake, the recommends culturally tailored education programs, such as community-led workshops, engaging religious leaders, and promoting male involvement strategies, alongside targeted interventions for underserved areas and strengthened healthcare systems to ensure equitable access. Implementing these evidence-based policies is essential for improving reproductive health and empowering women in Ethiopia.

## Background

Ethiopia faces significant challenges related to contraceptive use. This has far-reaching implications for maternal health, poverty reduction, and women’s empowerment. The country has a high fertility rate of 4.6 children per woman, which contributes to high maternal mortality and poverty, stemming from limited contraceptive uptake. ^1^ Only 25.9% (Mini-2019 EDHS) of women of reproductive age use or intend to use contraceptives. ^2^ This indicates that there is a high predictor of unmet need for family planning services, with a large majority (74.1%) do not use contraceptives. ^3, 4^ Several socio-cultural barriers and misconceptions contribute to this low contraceptive use. ^5– 7^ These include fatalistic beliefs, religious prohibitions, postpartum amenorrhea, spousal opposition, and concerns about side effects. The most common contraceptive non-use factors were fear of side effects/Health concerns, and breastfeeding. ^3^ Other factors include fatalistic beliefs, religious prohibitions, postpartum amenorrhea, and spousal opposition . ^8^ Factors such as wealth status, literacy, urban residency, husband’s education level, and regional disparities also play a role in influencing contraceptive use and intentions. ^9^ Regions like Somali and Afar exhibit significantly lower contraceptive uptake, partly due to nomadic lifestyles, limited health service access, and the strong influence of conservative religious beliefs compared to Addis Ababa. ^7^ Therefore, the intention to use contraceptives could be affected by both individual and community-level factors. ^10^ Addressing these multifaceted barriers through culturally sensitive policies is crucial for improving reproductive health outcomes and empowering women in Ethiopia. ^11^ Implementing socially acceptable and culturally sensitive strategies will increase contraceptive uptake, improve maternal health, and overall socio-economic development in Ethiopia. ^12^ Therefore, the Federal Ministry of Health (FMoH), regional health bureaus, and development partners should strengthen mass communication through media platforms and enhance health education delivered by health extension workers. Likewise, woreda health offices, in collaboration with community representatives, religious leaders, and local stakeholders, should prioritize the dissemination of family planning messages to foster informed decision-making and ultimately increase contraceptive use. ^8^

## Key findings

Data analyzed from both the 2016 EDHS and 2019 Mini-EDHS indicate that there is a low prevalence of contraceptive use intention among women of reproductive age (WRA). Only 25.9% of WRA reported intending to use contraceptives, while the majority (74.1%) did not use services. Among those not intending to use contraceptives, key reasons included fatalistic beliefs (19%), postpartum amenorrhea (14%), and religious prohibition (10%).

Multivariable analysis revealed several factors significantly associated with contraceptive use. Wealth status was a strong predictor; women in the richest wealth quintile were more likely to use contraceptives compared to the poorest (AOR = 3.61, 95% CI: 2.79-4.64). Literacy also played a significant role, with women able to read whole sentences having higher odds of contraceptive use than those with no education (AOR = 1.66, 95% CI; 1.31-2.11). Conversely, women whose husbands desired more children were less likely to use contraceptives (AOR = 0.81, 95% CI; 0.70-0.93), and rural women had lower odds of contraceptive use compared to urban women (AOR = 0.47, 95% CI; 0.37-0.60).

The Structural Equation Model (SEM) analysis further elucidated the direct and indirect effects of various factors on contraceptive use and intention. The analysis demonstrated a direct negative effect of fatalism, breastfeeding, husband opposition, postpartum amenorrhea, and education on contraceptive use and intention. Specifically, the direct effect of fatalism on contraceptive use and intention was (AOR = -0.050, P = 0.019). Interestingly, the SEM showed a direct negative effect of education on contraceptive use (AOR = -0.091), which may reflect subgroup effects or residual confounding and warrants further investigation. Furthermore, the SEM revealed that religious prohibition indirectly influences contraceptive use through its effect on fatalism. Religion has a direct negative effect on fatalism (AOR = -0.243, P < 0.0001). These findings suggest that religious teachings influence contraceptive behaviors indirectly by reinforcing fatalistic beliefs, highlighting the importance of engaging religious leaders to reshape community norms. Overall, the SEM highlights the complex interplay between socio-cultural factors like religious beliefs and individual factors like education in shaping contraceptive behavior in Ethiopia.

## Policy outcomes and implications

This policy brief aims to address the socio-cultural and economic barriers affecting contraceptive use in Ethiopia. By analyzing data from national health surveys, the study identifies key obstacles and proposes evidence-based interventions. The findings highlight the urgent need for policy adjustments to improve reproductive health outcomes and promote gender equity. This could include organizing dialogue forums with respected local elders and clergy to counter misconceptions and promote informed reproductive health choices. This policy brief aims to address the socio-cultural and economic barriers affecting contraceptive use in Ethiopia. Therefore, the policy implications suggest that without targeted interventions, contraceptive uptake will remain low, which further exacerbates maternal health risks and gender inequality.

## A call for evidence-based strategies to enhance contraceptive uptake in Ethiopia

Based on our analysis of the 2016 EDHS and 2019 Mini-EDHS data, we contend that significant policy adjustments are essential to address the persistent challenges in contraceptive use and intention within Ethiopia. Our findings highlight a concerningly low prevalence of contraceptive use intention among women of reproductive age (WRA) and reveal a complex interplay of socio-cultural, economic, and individual factors that impede effective family planning. To translate these research insights into tangible improvements in reproductive health outcomes, we strongly advocate for the following evidence-based policy strategies. Our research underscores the profound influence of socio-cultural norms and beliefs on contraceptive behavior. Therefore, we emphasize the need for health education programs meticulously tailored to address specific cultural contexts. Interventions should focus on dispelling prevalent misconceptions about contraception, providing accurate information about its benefits, and addressing barriers such as fatalistic beliefs and religious prohibitions through strategic engagement with the community and religious leaders.

We assert that empowering women is paramount to improving contraceptive uptake. Policies should prioritize initiatives that enhance women’s access to education and economic opportunities, thereby increasing their autonomy in family planning decision-making. Addressing gender inequalities must be a central focus, as our data indicates that these inequalities significantly constrain women’s ability to make informed reproductive choices.

Our findings also highlight the crucial role of male partners in contraceptive decision-making. We therefore recommend policies that actively promote male involvement in family planning. Interventions should target men to address their misconceptions, foster open communication within couples, and encourage shared responsibility in family planning.

We stress the importance of tailored interventions for specific populations that exhibit low contraceptive use. This includes targeted strategies for regions such as Somali and Afar, which demonstrate significantly lower utilization rates. These interventions must be culturally sensitive, address region-specific barriers, and ensure that family planning services are accessible and appropriate for the unique needs of these communities. We also emphasize the need for focused efforts to reach women in rural areas and those from lower socioeconomic backgrounds, who face disproportionate challenges in accessing and utilizing contraceptives.

We underscore the need for a robust and accessible health system to support contraceptive uptake. This includes ensuring a reliable need-based supply chain for contraceptives, providing comprehensive training for healthcare providers, and expanding service delivery points, particularly in underserved areas. Community health workers and mobile clinics are essential tools to extend services to remote populations and overcome geographical barriers.

To ensure accountability and effectiveness of policy interventions, we advocate for the establishment of rigorous data monitoring and evaluation systems. This includes systematic data collection on contraceptive use, reasons for non-use, and associated factors, with disaggregation by key variables such as age, education, wealth, and region. Such data will enable policymakers to track progress, identify areas requiring further attention, and make evidence-based adjustments to interventions.

In conclusion, we firmly believe that the implementation of these evidence-driven policy recommendations is critical to achieving meaningful improvements in contraceptive uptake, reducing unintended pregnancies, and ultimately enhancing the health and well-being of women and families across Ethiopia.

## Conclusion and Actionable Recommendations

The findings derived from the 2016 EDHS and 2019 Mini-EDHS data provide a critical foundation for reshaping family planning strategies in Ethiopia. Our analysis points to the urgent need to move beyond generalized approaches and embrace a nuanced understanding of the socio-cultural dynamics that shape contraceptive behavior. To achieve meaningful progress, we must prioritize interventions that not only address access and availability but also directly confront the deep-seated beliefs and norms that hinder contraceptive uptake.

To address these challenges, a comprehensive, community-based approach to family planning is essential. Culturally tailored education programs should be implemented to dispel myths and provide accurate information about contraceptive methods, ensuring that individuals have the knowledge needed to make informed reproductive choices. These educational initiatives should be delivered through schools, media, and community outreach programs to effectively reach diverse audiences.

Moreover, male engagement strategies must be developed to encourage men’s active participation in family planning decisions, fostering shared responsibility between partners. Programs that promote couple-based counseling, peer-led discussions, and positive role modeling can help shift traditional gender norms that often place the burden of contraception solely on women.

In addition, community-based interventions should be strengthened, particularly in underserved and rural regions where access to contraceptive services remains limited. Leveraging local health workers and community leaders can facilitate discussions, offer contraceptive counseling, and improve access to family planning services. By incorporating trusted figures within communities, resistance to modern contraceptive methods can be reduced, and acceptance can be increased.

At the same time, the healthcare system must be enhanced by ensuring a reliable supply chain for various contraceptive options and integrating family planning services into primary healthcare. This includes training healthcare providers to offer client-centered counseling and expanding service delivery points through mobile clinics and pharmacies. Revitalizing Ethiopia’s Health Extension Program requires updated training materials on family planning, increased budgetary support, and strengthened supervision and accountability frameworks. Strengthening supply chain management will help address stockouts and ensure that contraceptives remain consistently available to those who need them.

Furthermore, policy advocacy efforts should be intensified to support legislative reforms and secure increased funding for reproductive health programs. Engaging policymakers and stakeholders in prioritizing family planning within national health agendas will create a supportive environment for expanding contraceptive access. Legislative reforms that address structural barriers, along with sustained financial investments, will be crucial for long-term success.

Therefore, we issue a call for a paradigm shift in family planning programming. This paradigm shift could begin with stakeholder mapping and engagement, followed by pilot testing culturally adapted interventions, and eventually, national scale-up based on lessons learned. This shift also entails a commitment to sustained, community-driven initiatives that foster open dialogue, challenge harmful norms, and empower individuals to make informed reproductive choices. It demands a collaborative effort involving policymakers, researchers, healthcare providers, and community stakeholders working together to translate research insights into actionable strategies. Incremental approaches have shown limited progress; thus, a more proactive and coordinated strategy is needed to overcome persistent barriers. Therefore, a bold and transformative approach is essential to improve contraceptive prevalence, enhance reproductive health, and secure a healthier future for Ethiopia.

## Declaration

### Ethical approval and consent to participate

The consent to participate was obtained from the research participants during the original data collection process. The Ethiopian Public Health Institute (EPHI) institution’s research ethics review committee has provided ethical approval. The original data were collected in confirmation of international and national ethical guidelines. The purpose of the current analysis was to send to the DHS organization, and permission to download and use the data was obtained from the DHS organization.

## Consent for publication

Consent for publication was not applicable as this study does not contain identifiable patient data.

## Author’s contribution

The corresponding author led the conceptualization, design, and analysis of this study, and was responsible for drafting, revising, and finalizing the manuscript. Both the corresponding author and Baheru M.T. made significant contributions to the critical review and substantial revision of the manuscript in response to reviewer comments, which included rephrasing the abstract, enriching the background with stakeholder context, reorganizing the findings, and refining the policy recommendations.

## Data Availability

The specific DHS datasets used in this analysis are available from the DHS Program website: DHS Program. Details regarding the particular survey and files used can be accessed through https://dhsprogram.com/data/available-datasets.cfm. All the necessary information is required for a reader or reviewer to access the data by the same means as the authors.
