# Supplementary material for: Addressing socio-cultural barriers to improve contraceptive use in Ethiopia
Source: F1000Res. 2025 Jun 17;14:442. Originally published 2025 Apr 16. [Version 2] doi: 10.12688/f1000research.163555.2 (PMC12485327; doi:10.12688/f1000research.163555.2)
Supplement: Peer review history

# Reviewer report: Reviewer response for version 2

**DOI:** 10.5256/f1000research.183493.r414542

**Published:** 2025-09-30

**Authors:** Sarwat Mumtaz

**Affiliations:** 1 Ziauddin University, Karachi, Pakistan

Nice work; appreciated. This manuscript examines the persistent challenges surrounding contraceptive use and intention in Ethiopia, drawing on data from the 2016 Ethiopian Demographic and Health Survey (EDHS) and the 2019 Mini-EDHS. Despite decades of family planning initiatives, contraceptive uptake remains low, with only 25.9% of women of reproductive age reporting use or intention to use contraceptives, leaving a substantial unmet need. Key barriers include fatalistic beliefs, religious prohibitions, spousal opposition, misconceptions about side effects, and limited access to health services, particularly in rural and pastoralist regions such as Somali and Afar.

 Multivariable and Structural Equation Model (SEM) analyses highlight the complex interplay of socio-cultural, economic, and demographic factors. Wealth, literacy, and urban residency were strong positive predictors of contraceptive use, whereas fatalism, religious influence, spousal opposition, and postpartum amenorrhea negatively shaped behaviors and intentions. The findings also reveal indirect pathways, with religious teachings reinforcing fatalistic beliefs that suppress contraceptive adoption.

 Based on these insights, the manuscript calls for a paradigm shift in Ethiopia’s family planning strategy. Evidence-based, culturally tailored interventions are needed to address misconceptions, foster male engagement, and strengthen community-based delivery. Health system improvements — including reliable contraceptive supply chains, expanded service delivery, and revitalization of the Health Extension Program — are equally critical. Policy advocacy, legislative reform, and sustainable financing must support these initiatives to ensure scale-up and long-term impact. In conclusion, the study underscores that improving contraceptive uptake in Ethiopia requires a comprehensive, multi-level approach that addresses both socio-cultural barriers and systemic health service gaps. Implementing these strategies is essential for enhancing reproductive health, reducing maternal mortality, and advancing gender equity and socio-economic development.

 Overall, the manuscript is clear and very comprehensive with clear, balanced, and well-justified recommendations. However, there are some areas to polish. For example; (less repetition, more prioritization, and sharper action steps). Avoid redundancy; you repeat phrases like “comprehensive, community-based approach” and “paradigm shift”. They’re powerful but could be streamlined. All recommendations sound equally important but be more actionable. Some phrases are broad (e.g., “challenge harmful norms”). Policymakers respond better to concrete actions (e.g., “train 500 health extension workers in culturally tailored family planning counseling”).

Does the paper provide a comprehensive overview of the policy and the context of its implementation in a way which is accessible to a general reader?

Yes

Is the discussion on the implications clearly and accurately presented and does it cite the current literature?

Yes

Are the recommendations made clear, balanced, and justified on the basis of the presented arguments?

Yes

Reviewer Expertise:

Sexual and reproductive health, maternal health, quality assurance in healtcare

I confirm that I have read this submission and believe that I have an appropriate level of expertise to confirm that it is of an acceptable scientific standard.

# Reviewer report: Reviewer response for version 2

**DOI:** 10.5256/f1000research.183493.r392790

**Published:** 2025-06-28

**Authors:** Maretalinia Maretalinia

**Affiliations:** 1 Mahidol University, Salaya, Nakhon Pathom, Thailand

The updated version of the manuscript is well revised. There is no additional comment and feedback from my side

Does the paper provide a comprehensive overview of the policy and the context of its implementation in a way which is accessible to a general reader?

Yes

Is the discussion on the implications clearly and accurately presented and does it cite the current literature?

Partly

Are the recommendations made clear, balanced, and justified on the basis of the presented arguments?

Partly

Reviewer Expertise:

Multivariate analysis, women of reproductive age health, contraceptive use, longitudinal study

I confirm that I have read this submission and believe that I have an appropriate level of expertise to confirm that it is of an acceptable scientific standard.

# Reviewer report: Reviewer response for version 1

**DOI:** 10.5256/f1000research.179927.r383831

**Published:** 2025-05-29

**Authors:** Maretalinia Maretalinia

**Affiliations:** 1 Mahidol University, Salaya, Nakhon Pathom, Thailand

Abstract:

 The abstract does not adequately reflect the content of the full manuscript. It should summarize not only the study findings but also highlight the policy relevance and the broader context of socio-cultural barriers. As this manuscript is submitted as a policy brief, the abstract must be reframed to present a concise synthesis of existing knowledge, stakeholder perspectives, and implications for policy and practice.

 Background:

 The background section lacks clarity on the key stakeholders involved in this issue. It is important to specify whether ethnic group leaders, religious leaders, or community figures play influential roles in shaping contraceptive use. Furthermore, there is no mention of existing literature discussing cultural or religious prohibitions against contraception in Ethiopia. Given that this is a policy brief, a broader foundation of literature—including national policy documents, religious doctrines, and cultural narratives—should be integrated to support the analysis.

 Key Findings:

 This section resembles the structure and content of a research article, focusing on original data, statistical analysis, and odds ratios. However, in a policy brief, the key findings should summarize insights drawn from a synthesis of existing literature, policies, and stakeholder inputs. For example, referencing religious or ethnic group guidelines, or summarizing community attitudes documented in prior studies, would provide a stronger foundation for policy analysis.

 Policy Outcomes and Implications:

 The policy outcomes and implications section is underdeveloped. It primarily reiterates research findings without offering clear or actionable policy directions. Recommendations should be explicitly stated and linked to relevant stakeholders, such as ethnic leaders, religious authorities, and frontline health workers. Since community beliefs and norms are deeply embedded in social structures, proposed interventions must emphasize education, information dissemination, and communication strategies that actively engage these stakeholders. This would ensure culturally sensitive and community-supported policy implementation

Does the paper provide a comprehensive overview of the policy and the context of its implementation in a way which is accessible to a general reader?

Yes

Is the discussion on the implications clearly and accurately presented and does it cite the current literature?

Partly

Are the recommendations made clear, balanced, and justified on the basis of the presented arguments?

Partly

Reviewer Expertise:

Multivariate analysis, women of reproductive age health, contraceptive use, longitudinal study

I confirm that I have read this submission and believe that I have an appropriate level of expertise to confirm that it is of an acceptable scientific standard, however I have significant reservations, as outlined above.

# Response

**Published:** 2025-05-29

**Authors:** Tadesse Shiferaw Chekol

**Affiliations:** One Health Research Division, Armauer Hansen Research Institute, Addis Ababa, Addis Ababa, Ethiopia

Dear Reviewer,

 I sincerely thank you for your thoughtful and constructive feedback on my manuscript. I appreciate the time and effort you invested in reviewing this work and for approving it with reservations. Your comments have been invaluable in refining the clarity, relevance, and policy orientation of the manuscript. Below, I address each of your comments point-by-point and describe the corresponding revisions made to the manuscript.

 1. Abstract: Reframing to Reflect Policy Brief Format

 Thank you for this important observation. I have revised the abstract to align with the expectations of a policy brief. The new abstract provides a concise synthesis of key findings, contextual socio-cultural barriers, stakeholder perspectives, and clear policy implications. It now emphasizes the broader public health and gender equity context, as well as the relevance of the findings to national development and health policy.

 2. Background: Clarity on Stakeholders and Broader Literature

 I appreciate this insightful recommendation. The background section has been expanded to specify the influential roles played by religious leaders, ethnic elders, and community figures in shaping reproductive behaviors. I have also integrated references to relevant literature and national policy documents, as well as cultural and religious narratives that contribute to contraceptive use dynamics in Ethiopia. These additions enhance the foundation for the policy analysis that follows.

 3. Key Findings: Synthesis Over Statistical Detail

 Thank you for this helpful clarification. I have revised the "Key Findings" section to reflect a broader synthesis of insights from national surveys, literature, and community-level narratives, rather than emphasizing statistical outputs. While relevant data from the EDHS have been retained to ground the findings, they are now discussed in a narrative, policy-relevant format. Technical terms such as odds ratios have been removed to improve readability and accessibility for general policy audiences.

 4. Policy Outcomes and Implications: Specific, Action-Oriented Recommendations

 This comment is well taken. I have significantly revised the “Policy Outcomes and Implications” section to include specific, actionable recommendations. These now clearly identify and target relevant stakeholders, such as religious leaders, ethnic elders, health extension workers, and community influencers. The recommendations emphasize culturally grounded education campaigns, male engagement, gender empowerment strategies, and improved health system support. I have also included examples of communication strategies and stakeholder engagement mechanisms to support effective policy implementation.

 General Assessment Questions

 Thank you for Q1. I am pleased that the manuscript was found to be accessible to a broad readership.

 In response to Q2, I have expanded the discussion of implications by integrating current literature on socio-cultural influences on family planning in Ethiopia. Citations now include religious perspectives, local cultural norms, and policy frameworks relevant to reproductive

 For Q3, I have revised the recommendations to ensure they are directly aligned with the identified barriers and are presented in a clear and balanced manner. Each recommendation is now justified by the findings and supported by current evidence.

 Once again, I thank you for your valuable comments, which have significantly enhanced the policy relevance and practical utility of the manuscript.

# Reviewer report: Reviewer response for version 1

**DOI:** 10.5256/f1000research.179927.r381793

**Published:** 2025-05-24

**Authors:** Augustus Osborne

**Affiliations:** 1 Njala University, Freetown, Sierra Leone

General Comments

 The manuscript provides a timely and relevant analysis of socio-cultural barriers to contraceptive use in Ethiopia, drawing on data from the 2016 EDHS and 2019 Mini-EDHS. The study highlights critical issues such as fatalistic beliefs, religious prohibitions, and spousal opposition, which are significant obstacles to family planning. The policy recommendations are well-intentioned and align with the findings. However, the manuscript would benefit from clearer methodological details, deeper contextualization, and more nuanced discussion of limitations. Below are specific comments and suggestions for improvement.

 Specific Comments by Section 

 1. Abstract

 - The abstract succinctly summarizes the study’s objectives, methods, and key findings. However, it could be strengthened by briefly mentioning the structural equation modeling (SEM) results, as this is a key methodological contribution. 

 - The policy recommendations are listed but could be more specific. For example, instead of "culturally tailored education programs," briefly note what such programs might entail (e.g., community-led workshops, religious leader engagement). 

 2. Background 

 - The background effectively sets the stage for the study but could better contextualize Ethiopia’s socio-cultural landscape. For instance, it would be helpful to briefly explain why regions like Somali and Afar have lower contraceptive use compared to Addis Ababa (e.g., nomadic lifestyles, stronger religious conservatism). 

 - The citation of the fertility rate (4.6 children per woman) and contraceptive use intention (25.9%) is clear, but the manuscript should clarify whether these figures are from the 2016 EDHS, 2019 Mini-EDHS, or another source. 

 3. Key Findings

 - The multivariable analysis and SEM results are presented clearly, but the interpretation could be deepened. For example, the direct negative effect of education on contraceptive use (AOR = -0.091) seems counterintuitive and warrants further discussion. Is this due to confounding factors, or does it reflect a specific subgroup? 

 - The SEM analysis mentions indirect effects (e.g., religious prohibition influencing contraceptive use through fatalism), but the manuscript could better explain the practical implications of these pathways. How might policymakers leverage this insight? 

 4. Policy Outcomes and Implications 

 - The policy recommendations are comprehensive but somewhat repetitive across sections. Consolidating them into a single, structured list (e.g., ranked by feasibility or impact) would improve clarity. 

 - The call for "culturally sensitive" interventions is appropriate but vague. The manuscript should provide concrete examples, such as partnering with local religious leaders to address misconceptions or using community health workers to deliver tailored messaging. 

 - The recommendation to "enhance healthcare systems" is critical but lacks specificity. Suggesting practical steps (e.g., training providers in client-centered counseling, expanding mobile clinics) would strengthen this section. 

 5. Actionable Recommendations 

 - This section overlaps significantly with the "Policy Outcomes and Implications." Consider merging the two or distinguishing them more clearly (e.g., one for high-level policy, the other for grassroots implementation). 

 - The suggestion to "revitalize the Health Extension Program" is excellent but needs elaboration. What specific improvements are needed (e.g., increased funding, better training)? 

 6. Conclusion and Call to Action 

 - The conclusion is compelling but somewhat hyperbolic (e.g., "The time for incremental change has passed"). While the urgency is justified, the tone could be moderated to maintain academic rigor. 

 - The call for a "paradigm shift" is inspiring but would benefit from a clearer roadmap. What specific steps should stakeholders take first? 

 7. Ethical Approval and Data Availability

 - The ethical statement is clear and appropriate. However, the data availability section could be more specific. Instead of "details can be provided upon request," include a direct link to the DHS Program website or the specific survey used. 

 8. References 

 - The references are relevant but somewhat limited. Including more recent studies (e.g., post-2021) on socio-cultural barriers in Ethiopia or similar contexts would strengthen the literature review.

Does the paper provide a comprehensive overview of the policy and the context of its implementation in a way which is accessible to a general reader?

Partly

Is the discussion on the implications clearly and accurately presented and does it cite the current literature?

Partly

Are the recommendations made clear, balanced, and justified on the basis of the presented arguments?

Partly

Reviewer Expertise:

Public Health

I confirm that I have read this submission and believe that I have an appropriate level of expertise to confirm that it is of an acceptable scientific standard, however I have significant reservations, as outlined above.
